# NUTRARET: Effect of 2-Year Nutraceutical Supplementation on Redox Status and Visual Function of Patients With Retinitis Pigmentosa: A Randomized, Double-Blind, Placebo-Controlled Trial

**DOI:** 10.3389/fnut.2022.847910

**Published:** 2022-03-21

**Authors:** Lorena Olivares-González, David Salom, Emilio González-García, David Hervás, Natalia Mejía-Chiqui, Mar Melero, Sheyla Velasco, Bianca Tabita Muresan, Isabel Campillo, Nieves Vila-Clérigues, Eduardo López-Briz, Juan Francisco Merino-Torres, José María Millán, José Miguel Soriano Del Castillo, Regina Rodrigo

**Affiliations:** ^1^Pathophysiology and Therapies for Vision Disorders, Principe Felipe Research Center (CIPF), Valencia, Spain; ^2^Joint Research Unit on Rare Diseases CIPF-Health Research Institute Hospital La Fe (IIS-La Fe), Valencia, Spain; ^3^Department of Ophthalmology, Manises Hospital, Manises, Spain; ^4^Center for Biomedical Network Research on Rare Diseases (CIBERER), Madrid, Spain; ^5^Department of Neuroscience, Manises Hospital, Manises, Spain; ^6^Department of Applied Statistics, Operations Research and Quality, Universitat Politècnica de València, Valencia, Spain; ^7^Service of Pharmacy, La Fe University and Polytechnic Hospital, Valencia, Spain; ^8^Service of Endocrinology and Nutrition, University General Hospital, Valencia, Spain; ^9^Service of Endocrinology and Nutrition, La Fe University and Polytechnic Hospital, Valencia, Spain; ^10^Joint Research Unit on Endocrinology, Nutrition and Clinical Dietetics UV-IIS La Fe, Valencia, Spain; ^11^Molecular, Cellular and Genomic Biomedicine, IIS-La Fe, Valencia, Spain; ^12^Food & Health Laboratory, Institute of Materials Science, University of Valencia (UV), Valencia, Spain; ^13^Department of Physiology, University of Valencia (UV), Valencia, Spain

**Keywords:** retinitis pigmentosa, oxidative damage, nutraceuticals, antioxidants, visual function

## Abstract

Oxidative stress plays a major role in the pathogenesis of retinitis pigmentosa (RP). The main goal of this study was to evaluate the effect of 2-year nutritional intervention with antioxidant nutraceuticals on the visual function of RP patients. Secondly, we assessed how nutritional intervention affected ocular and systemic redox status. We carried out a randomized, double-blind, placebo-controlled study. Thirty-one patients with RP participated in the study. RP patients randomly received either a mixture of nutraceuticals (NUT) containing folic acid, vitamin B6, vitamin A, zinc, copper, selenium, lutein, and zeaxanthin or placebo daily for 2 years. At baseline and after 2-year of the nutritional supplementation, visual function, dietetic-nutritional evaluations, serum concentration of nutraceuticals, plasma and aqueous humor concentration of several markers of redox status and inflammation were assessed. Retinal function and structure were assessed by multifocal electroretinogram (mfERG), spectral domain-optical coherence tomography (SD-OCT) and automated visual field (VF) tests. Nutritional status was estimated with validated questionnaires. Total antioxidant capacity, extracellular superoxide dismutase (SOD3), catalase (CAT), and glutathione peroxidase (GPx) activities, protein carbonyl adducts (CAR) content, thiobarbituric acid reactive substances (TBARS) formation (as indicator of lipid peroxidation), metabolites of the nitric oxide (NOX) and cytokine (interleukin 6 and tumor necrosis factor alpha) concentrations were assessed by biochemical and immunological techniques in aqueous humor or/and blood. Bayesian approach was performed to determine the probability of an effect. Region of practical equivalence (ROPE) was used. At baseline, Bayesian analysis revealed a high probability of an altered ocular redox status and to a lesser extent systemic redox status in RP patients compared to controls. Twenty-five patients (10 in the treated arm and 15 in the placebo arm) completed the nutritional intervention. After 2 years of supplementation, patients who received NUT presented better retinal responses (mfERG responses) compared to patients who received placebo. Besides, patients who received NUT showed better ocular antioxidant response (SOD3 activity) and lower oxidative damage (CAR) than those who received placebo. This study suggested that long-term NUT supplementation could slow down visual impairment and ameliorate ocular oxidative stress.

## Introduction

Retinitis pigmentosa (RP) is the most common disease among the group of inherited retinal dystrophies. It constitutes the largest single cause of inherited blindness in the developed world (1). RP is considered a rare disease (prevalence 1 in 4,000 worldwide). It causes a progressive and irreversible loss of vision that, in most studied models and patients, parallels photoreceptor cell death (rods and cones) (2, 3). As occurs in many rare diseases RP exhibits a high genetic and clinical heterogeneity. To date, more than 130 genes are known to cause different forms of RP either non-syndromic or syndromic (e.g., Usher syndrome) (4).

At cellular level, RP is characterized by progressive rod degeneration in initial stages and eventual cone degeneration in later stages. It is highly probable that cone degeneration is influenced by oxidative stress, the release of inflammatory molecules, and other toxic substances from rods and surrounding cells (5). Rod degeneration could be exacerbated by these substances. Besides, several environmental risks such as exposition to high oxygen tension (hyperoxia) or ultraviolet radiation can also affect photoreceptor survival. These cells are highly susceptible to free radicals because of their elevated metabolic rate.

Oxidative stress is defined as a disturbance caused by an imbalance between the free radical generation and the antioxidant system activity, promoting the cell damage. Antioxidants are molecules that safely interact with free radicals to neutralize them. The antioxidant system includes endogenous antioxidants such as antioxidant enzymes [intracellular/extracellular SOD, glutathione peroxidase (GPx), and catalase (CAT)], non-protein antioxidants [glutathione (GSH), lipoic acid, bilirubin, etc.] or minerals (selenium, zinc, and iron), among others. The antioxidant system also includes dietary exogenous antioxidants such as some vitamins, flavonoids, β-carotenes, etc. Endogenous and exogenous antioxidants would contribute to reduce oxidative stress in photoreceptor cells.

In animal models of RP, several evidence supports the oxidative stress hypothesis (6–9). For instance, the retina of these RP models showed low levels of GSH, downregulation of antioxidant effectors and high levels of nitric oxide or malondialdehyde, among others (6, 9, 10). Some authors also detected alterations in different markers of oxidative stress and antioxidant response in RP patients. For instance, we previously found an altered antioxidant-oxidant (redox) status in aqueous humor and peripheral blood of RP patients. In that study, we observed a lower ocular antioxidant response [total antioxidant capacity (TAC) and extracellular superoxide dismutase (SOD3) activity] and a higher blood oxidative stress [thiobarbituric acid reactive substances (TBARS)] in RP patients than in controls (11). We showed a strong association between a better ocular redox status and a better visual visual field (VF) in these patients.

Campochiaro et al. also showed ocular oxidative stress but no systemic oxidative stress in RP patients (12). More recently, Ertan et al. reported oxidative stress in peripheral blood of RP patients with alterations in thiol/disulfide homeostasis (13). In fact, treatment with different antioxidant formulations (9, 14–16) or overexpression of the endogenous antioxidant enzymes (12, 17), reduced oxidative stress and cone cell death in different mouse models of RP.

Nutraceuticals (combination of “nutrients” and “pharmaceuticals”) are natural substances from foods with physiological benefits. They can act upon multiple targets or process simultaneously including antioxidant defense machinery, inflammation, gene expression, cell proliferation, cell death, etc. (18, 19). They include trace elements (selenium, zinc, and copper), polyunsaturated fatty acids (PUFAs), carotenoids (xanthophylls and carotenes), polyphenols, saponins, vitamins (A, C, and B group), etc. Those nutraceuticals could be strategies against different retinal diseases including RP, retinopathy of prematurity, age-related macular degeneration (AMD) or diabetic retinopathy (20–25).

Recently, we have described that a mixture of antioxidant nutraceuticals (called NUT) ameliorated visual function and redox status of *rd10* mice, a model of recessive autosomal RP (26). Based on these findings, we proposed to evaluate the effect of NUT on visual function and redox status in RP patients after 2 years of nutritional intervention.

In this prospective, double-blind, placebo-controlled study, 31 patients with RP were randomized to receive two tablets/day of a nutritional supplement containing a mixture of carotenoids, vitamins and minerals or placebo for 2 years. The main outcome measures included multifocal electroretinogram (mfERG) responses, spectral domain-optical coherence tomography (SD-OCT), and VF. Ocular and systemic redox status were also assessed by determination of several oxidant and antioxidant markers in aqueous humor and blood. Additionally, the levels of the cytokines interleukin (IL)6 and tumor necrosis factor alpha (TNFα) were determined. A Bayesian approach showing the probability of being outside a specific range considered as practically no effect (region of practical equivalence, ROPE) was performed.

## Materials and Methods

### Chemicals and Reagents

Trichloroacetic acid (TCA, #8223420025), Na_2_CO_3_ (#S7795), and retinol (vitamin A, #R7632, as standard for UHPLC analysis), nitrate reductase (#10981249001) were purchased from Sigma-Aldrich (St. Louis, MO, United States). Pyridoxine HCl (vitamin B6, #31650-22), folic acid (vitamin B9, #30146-21), retinol acetate (#31640-22), selenium (#32445-25), sulfate zinc (#31689-27), and sulfate copper (#30444-27) were purchased from Fagron Iberica SAU (Barcelona, Spain). Lutein (#RDNU-20-339) and zeaxanthin (#RDNU-20-342) were purchased from Dynadis SARL (Auterve, France). Methanol, chloroform, acetonitrile, tetrahydrofuran and water LC-MS grade were obtained from Merck (Darmstadt, Germany) or Scharlab S.L. (Barcelona, Spain).

### Study Population

This study was performed in accordance with the Declaration of Helsinki. It was reviewed and approved (reference: 2013/0388) by the Medicaments Research Ethics Committee (CEIm) of La Fe University Hospital (Valencia, Spain). Management of personal data of the participants was in accordance with the Organic Law 15/1999 and the Regulation (EU) 2016/679 (GDPR). Collected data were anonymized.

Patients with various stages of RP were recruited from Retina Comunidad Valenciana-Asociación Afectados por Retinosis Pigmentaria (RETINACV), and the Service of Ophthalmology of Manises Hospital (Valencia, Spain) between June 2015 and April 2017 according to the following inclusion criteria: elevated final dark-adaptation threshold, retinal arteriolar narrowing, and a reduced and delayed electroretinograms (ERG), pigmentary alterations characteristic on the periphery of the retina, concentric decrease in VF; best-corrected visual acuity (BCVA) ≥20/100; total sensitivity score of the points of the VF ≥50 dB; cones amplitude in Ganzfeld ERG of 30-Hz ≥0.48 μV; over the age of 18; body mass index (BMI) ≤40 and weight ≥ to the fifth percentile for each age, sex, and height.

Individuals with atypical RP (RP paravenous, Refsum disease, and Retinitis Punctata Albescens); patients with profound congenital deafness or other ocular diseases like glaucoma, diabetic retinopathy, uveitis, posterior subcapsular cataracts >11% of lens area (P3 del LOCSIII); patients with antioxidant supplementation; pregnancy or allergy to the Compositae (Asteraceae) family of plants or tartrazine were excluded. Thirty-three RP patients were initially included in this study (Table 1). Twenty-six adult subjects with no confounding ocular or systemic disease (blood donors and nutritional evaluation) and 16 patients suffering from cataracts without any other ocular or systemic disease (aqueous humor donors) served as controls. Controls were recruited from the Ophthalmology Service of La Fe University Hospital (Valencia, Spain) and from the Biobank La Fe (Valencia, Spain).

**TABLE 1:** Characteristics of participants.

Characteristics	Controls (for blood samples)	Controls (for ocular samples)	RP
Males	10	7	18
Females	16	9	15
Age (year) Mean (SEM)	44 (3)	68 (5)	51 (2)
Smokers (n smokers/group)	6/26	–	7/33
Weight (kg)	70.6 (18.3)	–	71.1 (17.2)
Height (cm)	168 (8)	–	166 (8)
BMI (kg/m^2^)	24.9 (1.4)	–	25.6 (4.5)
Caloric intake (kCal/day)	1819 (93)	–	1,706 (38)
Protein intake (g/day)	78 (6)	–	72 (3)
Fat intake (g/day)	83 (7)	–	76 (4)
Carbohydrate intake (g/day)	176 (15)	–	175 (7)
Dietary fiber intake (g/day)	22 (2)	–	19 (1)
ORAC (μmol TE/g.day)	11,934 (1,644)	–	17,313 (1,951)
Vitamin A intake (μg/day)	760 (119)	–	729 (54)
Vitamin C intake (mg/day)	153 (22)	–	124 (11)
Vitamin E intake (mg/day)	8.9 (0.7)	–	7.9 (0.5)
Carotenoids intake (μg/day)	2,522 (455)	–	2,488 (293)
Zn intake (mg/day)	8.8 (0.7)	–	8.7 (0.4)
Fe intake (mg/day)	13.7 (1.2)	–	12.2 (0.7)
Se intake (mg/day)	145 (21)	–	88 (5)

*BMI, body mass index; TE, Trolox equivalents; Zn, zinc; Cu, copper; Se, selenium.*

### Study Design

**FIGURE 1:**
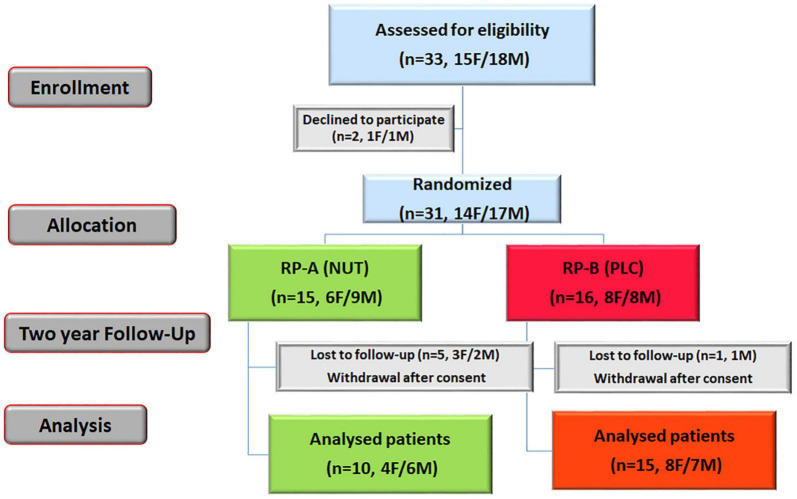
NUTRARET flow chart of the participants. This was a randomized, double-blind, placebo-controlled study that investigated the effectiveness of a mixture of nutraceuticals (NUT) in ameliorating redox status and visual function in patients with RP. Thirty-one eligible RP patients were randomized to NUT group (A) or placebo (PLC) group (B), and daily received capsules with NUT or PLC for 2 years. F, female; M, male.

Thirty-three patients with typical forms of RP were initially enrolled for basal analysis of redox status. After baseline data collection, two of them withdrew before starting NUT supplementation because they decline to participate by personal reasons. Finally, 31 of these patients with RP were randomized to parallel arms of the study, and received capsules containing either NUT or placebo (PLC) for a period of 2 years (Figure 1). Consecutive patients who met inclusion criteria and do not met exclusion criteria were randomized following a simple randomization procedure^1^ by means of balanced blocks of five. Pharmacy Department kept the code list and, after a phone call of investigators requesting treatment for a new patient, dispensed medication according to randomization list (group A, NUT arm; group B, PLC arm); this procedure assures the allocation concealment. The exact nature of product gave to the patients (NUT or PLC) was willing to divulge group assignment to the patient’s physician only in case of a medical emergency, but this option was not used along the study.

PLC and NUT–enriched capsules were identical in size and appearance to assure blindness of study. Oral supplementation with two enriched capsules provided 300 μg folic acid, 3 mg vitamin B6, 1.4 mg vitamin A, 16 mg zinc, 4 mg copper, 70 μg selenium, 16 mg lutein, and 38 mg zeaxanthin daily; excipient formula was based on microcrystalline cellulose (98.05%), colloidal anhydrous silica (1.95%). Matched PLC oral capsules contained tartrazine (E102, FD&C Yellow 5) to make contents similar in color to NUT-enriched capsules. The formulation of NUT was designed according to the Dietary Reference Intakes (DRI) for the Spanish Population for each compound (27) by Dr. José Miguel Soriano (Food & Health Laboratory, Institute of Materials Science, UV, Valencia, Spain).

All PLC and NUT-enriched capsules were prepared at clean rooms and labeled either A or B by the Department of Pharmacy (La Fe University and Polytechnic Hospital, Valencia, Spain) to mask study participants and research staff.

During informative talks and telephone calls we explained to the patients the instructions of the study. Patients were instructed to take two capsules per day with no recommended changes in their normal dietary practices and to visit every 2 months RETINA CV office or the hospital to receive capsules for the following months. RETINA CV actively participated in the distribution of the capsules. To assess patient adherence we used indirect methods: (i) we counted the number of bottles they collected every 2 months; (ii) we collected information from patients (self-reported) after each visit, or telephone call (e.g., how many bottles remained at home). Some patients decided to withdraw from the study. The reasons given for leaving the study were pregnancy (one participant), relocation (two participants), time conflicts because of full-time job and childcare (one participant), personal reason (one participant), non-compliance (aqueous humor sampling, one participant). Therefore, all of the dropouts were considered as missing completely at random (MCAR). Subjects were encouraged to report any adverse effects immediately. All subjects underwent a clinical survey that included specific parameters that might influence the outcome of the performed analyses, such as smoking habit (Table 1). No macular edema was observed in the participants.

Follow-up study visits were scheduled annually (three medical visits: V1, V2, and V3); follow-up also included telephone calls every 4 months from a nutritionist. At baseline (V1) and after 24 months (V3) participants received a complete eye examination (fundus eye, SD-OCT, automated VF, and mfERG responses). After 12 months (V2) participants received a routine eye examination (fundus eye and VFs). At annual visits (V2 and V3) and, during telephone calls, participants were required about adverse events or any other event of interests.

After randomization, blood and aqueous humor samples were collected at the beginning and at the end of the intervention at the hospital. For controls a 1-time blood or aqueous humor sample was drawn as described for study participants.

### Dietary Assessment and Dietary Antioxidant Capacity Estimation

For the dietetic-nutritional evaluation, two types of food surveys were used: (i) 24-h recall questionnaire conducted in triplicate, according to the European recommendations (28) and methodology (29) and, (ii) validated Spanish food frequency questionnaire (30). Each questionnaire provides detailed information about all foods, beverages, dietary supplements, tobacco and alcohol consumption, the presence of fruits, vegetables, unhealthy food, or signs of an antioxidant deficiency in the diet. Recipes were separated according to their ingredients.

To avoid confounding dietary antioxidant factors such as saffron, etc., the consumption of dietary antioxidant was assessed with the estimation of portion sizes from 24-h recall questionnaire, interviewed by nutritionists, coupling with a picture book, including country-specific dishes, with additional household and other relevant measurements. Daily intake of macronutrients (proteins, fat, carbohydrates, and fiber) and micronutrients such as vitamins (A, C, D, and E), carotenoids and minerals (copper, selenium, and, zinc) were estimated using the computer DIAL 3.0.0.5 program (31). Food consumption was expressed as g/person/day for macronutrients, mg/person/day for vitamins C, D, E, iron, and zinc or as μg/person/day for vitamin A, carotenoids, and selenium (Table 1). Values were expressed as mean ± standard error.

Apart from estimating vitamin and mineral intakes, we determined daily caloric intake as Kcal/day and the hydrophilic and lipophilic antioxidant capabilities of the diet using the oxygen radical absorption capacity (ORAC) scores of food (32). ORAC values were reported in mmol of Trolox equivalents per gram (TE/g). The antioxidant capacity of the diet describes the ability of antioxidants present in food to remove free radicals (33).

### Visual Assessment

A full ophthalmic examination from both eyes was performed at baseline and after 2 years of nutritional intervention including SD-OCT examinations, automated VF and mfERG responses at the Manises Hospital between June 2015 and November 2019.

SD-OCT imaging was done using the Cirrus HD-OCT device (Cirrus HD-OCT software version 6.0.0.599, Carl Zeiss Inc., Zena, Germany). All SD-OCT scans were performed by the same operator and both eyes were tested. A macular thickness map protocol with a macular cube 512 × 128 combo was used to obtain the mean macular thickness measurements in nine Early Treatment Diabetic Retinopathy Study (ETDRS) regions at baseline and after 2 years of supplementation. These nine regions were located in three rings with diameters of 1 (perifovea ring), 3 and 6 mm. The two last rings were divided into four quadrants: superior, inferior, temporal, and nasal. We obtained the mean retinal thickness measurements for these nine regions (μm).

The automated VF assessment was performed using a Humphrey automated VF Analyzer (Zeiss Inc., Zena, Germany) or perimeter. A standardized automated procedure 30-2 complete threshold with number V stimulus was used to measure retinal sensitivity in decibel (dB). The retinal sensitivity was represented in the four quadrants of the VF. The numbers in each quadrant indicate the threshold of stimulus intensity detected in dB. Typical normal values centrally are around 30 dB. Finally, the sum of all the values for the four quadrants (from both eyes) was calculated.

Electrophysiological tests were conducted to check retinal function. Thus, all patients with RP underwent mfERG analysis. Updated standards of the International Society for Clinical Electrophysiology of Vision (ISCEV) (34–40) were followed to obtain all recordings, using a Roland Consult equipment with the application software RETIscan 3.20/RETIport 32. mfERG was carried out with a CRT monitor with a central fixation point marked with a X across the entire screen. The stimulus array consisted of 103 hexagons, which change from black to white in a pseudo-random way. The patient was placed with a fixation angle on the screen of 30 min with the head resting on an adjustable chin cup, and a minimum of eight exploration cycles (at least 1 min each) and a maximum of 12 (until responses that could be assessed were obtained) were displayed.

The active HK-Loop electrodes were placed inside the conjunctival sac to prevent vision interferences. The surface reference electrodes were located on the external edge of each eye, and the ground electrode was placed on the forehead. The impedance of the electrodes was kept under 5 kΩ (with less than 20% difference between electrodes) using abrasive paste Everi^®^ from Spes Medica Srl (Genova, Italy) to clean the skin, applied with sterile gauze pads. The surface electrodes were filled with conductive paste Ten20^®^ Conductive and were fixated on the skin with Micropore™ Hipoalergenic tape from 3M (MN, United States). Pupils had been dilated with pupil diameter of at least 7 mm. Refraction was corrected with lenses to achieve that patients were able to fixate the screen properly.

The changes observed in the mfERG evaluated the central 30° of the retina following a standard distribution in quadrants. To analyze the mfERG responses from each eye, the results were grouped into quadrants. The sum of the P1 amplitudes and implicit time for P1 amplitudes of the four quadrants was presented in nV/deg^2^ and milliseconds (ms), respectively.

### Blood and Aqueous Humor Collection

Participants attended with 12 h fasting the hospital (Service of Ophthalmology). Blood samples were initially collected from 33 RP patients and 26 healthy subjects in heparinized (plasma) or non-heparinized tubes (serum). After sampling, tubes were placed in racks in vertical upright position in transportation boxes at 4°C. Boxes were transported to the laboratory at CIPF. Once in the laboratory samples were immediately registered with a code. Blood was processed within 2 h after collection. Samples were centrifuged for 10 min at 800 *g* or for 5 min at 2,000 *g* to obtain plasma or serum. Plasma and serum were stored in several aliquots at −80°C until biochemical determinations.

Aqueous humor were initially collected from 33 RP patients under sterile conditions in a surgical theater (Service of Ophthalmology). We applied one drop of povidone iodine before and after the anterior chamber was punctured using a 30-gauge needle. Antibiotic prophylaxis was subsequently administered for several days. Aqueous humor samples from 16 patients suffering from cataracts without any other ocular or systemic disease were collected with a 30-gauge needle just before the beginning of the cataract surgery and served as controls. The aqueous samples of controls were collected under the same conditions before cataract surgery began. Aqueous samples were registered with a code. Undiluted aqueous samples, of at least 100 μL, were collected from each patient, placed in sterile tubes, and aliquoted (25 μL/aliquot). Aliquot tubes were placed in transportation boxes with dry ice and transported to the laboratory at CIPF. Tubes were immediately stored at −80°C until use. All specimens were assayed to evaluate redox status. For each patient, aqueous humors were collected from the eye with the more severe retinopathy or alternatively from the eye with the worse visual acuity (VA).

For RP patients, sample collection was carried out two times: baseline (*t*0, *n* = 33), and at the end of the treatment (24 months, *t*1, *n* = 25). Freeze-thaw cycles were avoided. We always analyzed the samples at the same time with the same procedures to minimize technical errors.

### Determination of Serum Trace Elements

Frozen serum (0.1 mL) was deproteinized with 0.1 mL of TCA 7%, before analysis. The supernatant was centrifuged at 10.000 *g* for 5 min at 4°C. Supernatants were neutralized with NaCO_3_ 1M. Neutralized supernatants were used to determine zinc, copper and selenium concentrations using commercial kits. Zinc was quantified using a fluorometric kit (#ab176725, Abcam, Cambridge, United Kingdom) according to manufacturer’s instructions. Zinc bound to a probe with enhanced fluorescence at Ex/Em 485/525 nm. This probe presented a high increase in fluorescence in response to zinc.

Copper was also quantified using a fluorometric kit (#ab252901, Abcam, Cambridge, United Kingdom) according to manufacturer’s instructions. Copper ions catalyze a reaction that produces a highly fluorescent product (Ex/Em 395/480 nm). The reaction rate was proportional to the amount of Copper in serum.

Selenium was quantified using a colorimetric kit (#abx298910, Abbexa LTD., Cambridge, United Kingdom) following a slightly modified procedure of the manufacturer’s instructions. Selenium reacted with the reagents of the kit to produce a colored compound that was detected at 520 nm. Zinc and copper were expressed as μg/dL and selenium was expressed as μg/L.

### Sample Extraction and Ultra High Performance Liquid Chromatography-Tandem Mass Spectrometry Analysis

Extraction of polar and non-polar compounds from serum was achieved by a modification on the ultra high performance liquid chromatography (UHPLC) tandem mass spectrometry (MS/MS) method described by Hrvolová et al. (41). Frozen serum (0.1 mL) was treated with 0.2 mL of methanol, vortex mixed for 40 s and centrifuged at 12,000 g for 5 min. The organic upper layer was transferred into another tube. The precipitate was reconstituted in 0.2 mL of chloroform with water bath sonication for 5 min, vortex mixed for 40 s and centrifuged at 12,000 *g* for 5 min. Both organic upper layers were combined. The combined was filtered through a 0.22 μm PTFE filter. The filtrate was dried under a stream of nitrogen (N_2_) and lyophilized. The lyophilized was reconstituted in 0.1 mL of methanol (sample stock-1). An aliquot of 0.01 mL was transferred into an amber glass vial and diluted with 0.01 mL of a mixture of acetonitrile/water/tetrahydrofuran (THF) (3:3:4) and injected onto the UHPLC column. Serum samples and stock solutions were stored at −80°C.

Retinol (vitamin A), pyridoxine (vitamin B6), folic acid (vitamin B9), lutein, and zeaxanthin were detected and quantified using a UHPLC apparatus (Shimadzu, LCMS-8040) coupled to a MS/MS triple quadrupole equipped with electrospray ionization (ESI) operating in positive and negative ionization mode (Shimadzu, Kyoto, Japan) at the Institute of Health Research-INCLIVA (Valencia, Spain).

Ultra high performance liquid chromatography analysis was performed on reversed-phase chromatography with a C18 Kinetex Evo (3.0 × 150 mm, 2.6 μm, 100 Å) column (Phenomenex Technologies Inc., Torrance, CA, United States). All compounds were monitored in multiple reaction monitoring (MRM) mode. MRM transitions were achieved by a LabSolutions method development software (Shimadzu). Ion source conditions were optimized to attain the highest possible responses for all compounds. The MS source parameters were as follows: DL temperature of 250°C, heat block temperature of 400°C, nebulizing gas flow of 3 L/min, drying gas flow of 15 L/min.

Standard curves were generated by mixing solutions with varying concentrations of authentic standards in a mixture of acetonitrile/water/THF (3:3:4). Concentration ranges in the curves were from 0.1 to 0.5 ppm for retinol (retention time, Rt = 2.08 min), and from 0.5 to 8 ppm for pyridoxine (Rt = 1.12 min), folic acid (Rt = 1.71 min), lutein (Rt = 2.84 min), and zeaxanthin (Rt = 2.83 min). Limits of detection (LOD), limits of quantification (LOQ) and determination coefficient (*r*^2^) for each analyte were as follows: 0.02 ppm (LOD), 0.06 ppm (LOQ), 0.98 (*r*^2^) for retinol; 0.01 ppm (LOD), 0.01 ppm (LOQ), 0.99 (*r*^2^) for pyridoxine; 0.04 ppm (LOD), 0.13 ppm (LOQ), 0.99 (*r*^2^) for folic acid; 0.01 ppm (LOD), 0.03 ppm (LOQ), 0.99 (*r*^2^) for lutein; 0.01 ppm (LOD), 0.04 ppm (LOQ), 0.98 (*r*^2^) for zeaxanthin. Identification of compounds was performed by co-elution with authentic standards and matched relative intensity of MS/MS transitions.

The following MRM transitions and mass ions were used for quantification of each analyte which matched with standards: m/z 269.0 > 95.25, 93.10 for retinol, m/z 169.90 > 152.05, 133.95 for pyridoxine, m/z 440.20 > 311.15, 132.15 for folic acid, m/z 568.0 > 476.35, 338.35 for lutein and m/z 568.0 > 476.35, 119.10 for zeaxanthin. The most intense fragment ion was chosen as quantifier, and the second most abundant as qualifier. Retinol, lutein and zeaxanthin were expressed as mg/L (ppm). We were not able to quantify folic acid and vitamin B6 in sera samples.

### Determination of Antioxidant and Oxidant Markers

Ocular redox status was evaluated by measuring TAC, activities of SOD3, CAT, and GPx and CAR (indicator of protein carbonylation); indicator of protein carbonylation) in frozen aqueous humor samples. Blood redox status was evaluated by measuring TAC, activities of SOD3, CAT, and GPx, CAR, nitrites (stable end-product of NO) and nitrates (NOX) levels, and TBARS (as indicator of lipid peroxidation) formation in frozen plasma. We minimized the re-use of thawed aliquots, especially for the measurement of enzymatic activities and TAC.

Specifically, TAC was measured by detecting the oxidation of 2,2′-azino-di(3—ethylbenzothiazoline sulfonate) (ABTS) by metmyoglobin (absorbance at 405 nm) (#709001, Cayman Chemical, Ann Arbor, MI, United States). TAC levels were expressed as μmol of trolox equivalent/mL.

Determination of SOD3 or extracellular SOD activity were based on the dismutation of superoxide oxygen and hydrogen peroxide with a commercial kit. A tetrazolium salt reacted with superoxide radicals generated by xanthine oxidase and hypoxanthine and the formazan dye was colorimetrically measured (absorbance al 450 nm) (#706002, Cayman Chemical, Ann Arbor, MI, United States). SOD3 activity was expressed as U/mL.

Determination of CAT activity was based on the reaction of CAT with methanol in the presence of H_2_O_2_. The formaldehyde produced was measured colorimetrically with 4-amino-3-hydrazino-5-mercapto-1,2,4-triazole (absorbance at 540 nm) (#707002, Cayman Chemical, Ann Arbor, MI, United States). CAT activity was expressed as nmol of formaldehyde/minutes.mL.

Determination of GPx activity was measured indirectly by a coupled reaction with glutathione reductase (GR). Oxidized glutathione produced by GPx was recycled to its reduced state by GR and NADPH. The oxidation of NADPH to NADP^+^ was colorimetrically measured (absorbance at 340 nm) (#703102, Cayman Chemical, Ann Arbor, MI, United States). GPx activity was expressed as nmol/minutes.mL.

Lipid peroxidation was evaluated by measuring the formation of TBARS [including malondialdehyde (MDA) which was formed as a byproduct of lipid peroxidation] (Cayman Chemical, Ann Arbor, MI, United States). TBARS levels were expressed as nmol of MDA/mL.

Nitric oxide metabolites levels were measured by nitrate reductase and Griess reaction (42). NOX levels were expressed as nmol/mL.

Protein carbonyl adducts content was measured using fluorescein-5-thiosemicarbazide (FTC), a fluorescent probe which covalently reacted with oxidized residues on proteins. FTC generated stable fluorometric signal which was monitored (EX/Em 485/535 nm) (#ab235631, Abcam, Cambridge, United Kingdom). CAR content was expressed as pmol/mL.

### Determination of Tumor Necrosis Factor Alpha and Interleukin 6 in Blood

IL6 and TNFα concentrations were measured in frozen serum using enzyme-linked immunosorbent assay (ELISA) systems. We used an aliquot for this purpose. In particular, TNFα and IL6 protein levels were estimated with a high sensitivity ELISA kit (#BMS223HS and #BMS213HS, Human IL-6 High Sensitivity ELISA, Thermo Fisher Scientific, Waltham, MA, United States), according to the manufacturers’ instructions. Serum TNFα and IL6 levels were expressed as pg/mL.

### Statistical Analyses

All statistical analyses were done using R software (version 2.15.3) (Foundation for statistical computing, Vienna, Austria). A Bayesian analytical approach was carried out to calculate the probability that the nutritional intervention was superior or not, and the probability that a clinically relevant difference existed between groups. The ROPE (43, 44) and the credible interval (CrI) were calculated for each parameter. ROPE is an approach that considers that an effect is present, not only when it has a large probability of differing from zero, but when it has a large probability of being outside a specific range that can be considered as “practically no effect.” This range is called ROPE. This approach ensures that results are scientifically relevant, because it discards effects that could be considered negligible. We used Bayesian inference and ROPEs in order to be able to compute direct probabilities of the hypotheses being tested instead of potentially misleading *P*-values. Therefore we did not present *P*-values (frequentist approach) except for the main visual outcomes. The Figures except for Figures 1,5 are only descriptive, a way to visualize the data (mean and SD). They should not be interpreted as the results of the statistical analysis. Especially in this study, where the different observations were non-independent and where the models were multivariable for controlling differences among groups regarding important covariates. These aspects cannot be corrected in a figure. Statistical analysis is shown in Tables 2, 3, 7, 8.

**TABLE 2:** Bayesian analysis of estimated dietary intakes and blood nutrients in controls and RP patients.

	ES	95% CrI	Pr of RP < C	ROPE	Pr of RP > C
**Dietary antioxidant intake**					
ORAC	0.302	−0.188;0.780	0.055	0.141	0.804
Vitamin A	0.136	−0.217;0.486	0.129	0.201	0.669
Vitamin C	–0.137	−0.483;0.200	0.670	0.204	0.125
Vitamin E	–0.134	−0.368;0.107	0.783	0.144	0.072
Carotenoids	0.19	−0.423;0.812	0.170	0.223	0.606
Se	–0.428	−0.686;−0.163	0.998	0.0017	0.0002
Fe	–0.110	−0.325;0.108	0.747	0.164	0.088
Zn	–0.251	−1.755;1.212	0.484	0.271	0.244
**Serum nutrients**					
Retinol	0.002	−0.064;0.068	0.371	0.2077	0.421
Lutein	0.787	0.33;1.233	0.000	0.001	0.999
Zeaxanthin	0.822	0.246;1.395	0.0005	0.003	0.9965
Zn	–0.996	−6.933;4.690	0.0623	0.927	0.011
Cu	0.405	−5.325;6.129	0.108	0.727	0.165
Se	0.362	−5.407;6.37	0.008	0.977	0.015

*ES, estimate; Crl, credible interval; Pr, probability; RP, patients with retinitis pigmentosa: C, controls; ROPE, region of practical equivalence; ORAC, oxygen radical absorbance capacity; Fe, iron; Zn, zinc; Cu, copper; Se, selenium.*

**TABLE 3:** Bayesian analysis of baseline redox status and cytokines in controls and RP patients.

	ES	95% CrI	Pr of RP < C	ROPE	Pr of RP > C
**Ocular redox status**					
TAC	–0.693	−1.115;−0.277	0.999	5 × 10^–4^	0.0002
SOD3	–0.757	−1.261;−0.269	0.997	0.002	0.0007
CAT	–0.090	−0.704;0.526	0.547	0.120	0.334
GPX	–0.028	−0.521;0.473	0.474	0.144	0.381
CAR	0.68	0.078;1.283	0.008	0.015	0.976
**Blood redox status**					
TAC	0.056	−0.022;0.131	0.041	0.077	0.880
SOD3	–0.151	−0.292;−0.01	0.962	0.029	0.008
CAT	–0.239	−0.513;0.041	0.902	0.076	0.021
GPX	–0.989	−6.42;4.488	0.332	0.544	0.123
CAR	–0.007	−0.193;0.18	0.408	0.247	0.345
TBARS	0.228	−0.099;0.575	0.445	0.102	0.853
NOX	0.043	−0.601;0.672	0.319	0.234	0.446
**Blood cytokines**					
TNFα	0.425	0.22;0.631	0.000	0.0002	0.999
IL6	–0.263	−0.848;0.34	0.726	0.151	0.122

*ES, estimate; Crl, credible interval; Pr, probability; RP, patients with retinitis pigmentosa; C, controls; ROPE, region of practical equivalence; TAC, total antioxidant capacity; SOD3, extracellular superoxide dismutase; CAT, catalase; GPX, glutathione peroxidase; CAR, protein carbonyl adducts; TBARS, thiobarbituric acid reactive substances; NOX, nitrites and nitrates.*

## Results

### Antioxidant Nutritional Evaluation in Patients With Retinitis Pigmentosa

We analyzed the intake of dietary antioxidants and baseline serum levels of antioxidants in RP patients and controls. We included gender and age as confounding factors in the analysis. We obtained nutritional information from 33 patients with typical forms of RP and 26 controls (Table 1). Dietary antioxidants are complex mixtures of hundreds of compounds including micronutrients such as the vitamins A, C, D, and E and the minerals copper, zinc, or selenium, among others.

In this study we used the DIAL software to estimate the intake of vitamins and minerals and the dietary antioxidant intake by calculating ORAC scores. As shown in Tables 1, 2 the estimated intake of vitamins A, C, and E, carotenoids and minerals iron and zinc seemed to be similar between both groups. However, the probability that the estimated intake of selenium was higher in controls than in RP patients was very high (99.8%). But the probability that RP patients had higher values of ORAC than controls was 80.4%.

The anthropometric data of weight and height and daily caloric intake were similar in both groups. Estimated carbohydrate, fat, and protein intake were indistinguishable in both groups (Table 1).

**FIGURE 2:**
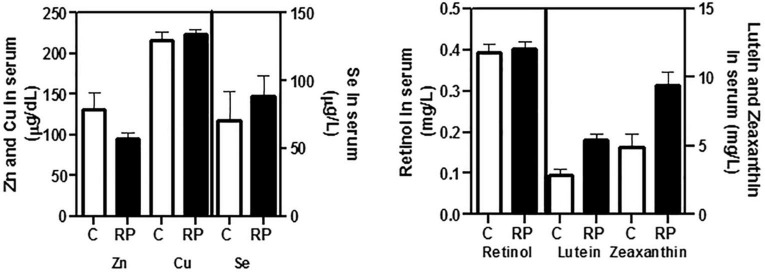
Baseline concentrations of antioxidant nutrients in serum of controls and RP patients. Retinol, vitamin B6, folic acid (vitamin B9), lutein, and zeaxanthin were determined by ultra high performance liquid chromatography (UHPLC). Vitamins B6 and B9 were not detectable by UHPLC in the samples. Trace elements zinc (Zn), copper (Cu), and selenium (Se) were determined with fluorometric commercial kits. All data were plotted in bar graph with standard error bars. C, control subjects; RP, patients with retinitis pigmentosa.

As shown in Figure 2, serum retinol, copper, zinc, and selenium concentrations were similar in both groups. However, the probability that lutein and zeaxanthin concentrations were higher in RP patients than in controls was very high (99.9 and 99.7%, respectively) (Table 2).

### Imbalanced Redox Status in Patients With Retinitis Pigmentosa

Thirty-three RP patients were initially enrolled for basal analysis of ocular and blood redox status. We enrolled different controls for ocular (16 controls) and blood (26 controls) redox status (Table 1). We were not able to obtain both samples from the same group of controls. We measured baseline aqueous humor levels of TAC, SOD3, CAT, and GPx activities and CAR, a marker of oxidative damage in proteins (45), before starting NUT supplementation.

**FIGURE 3:**
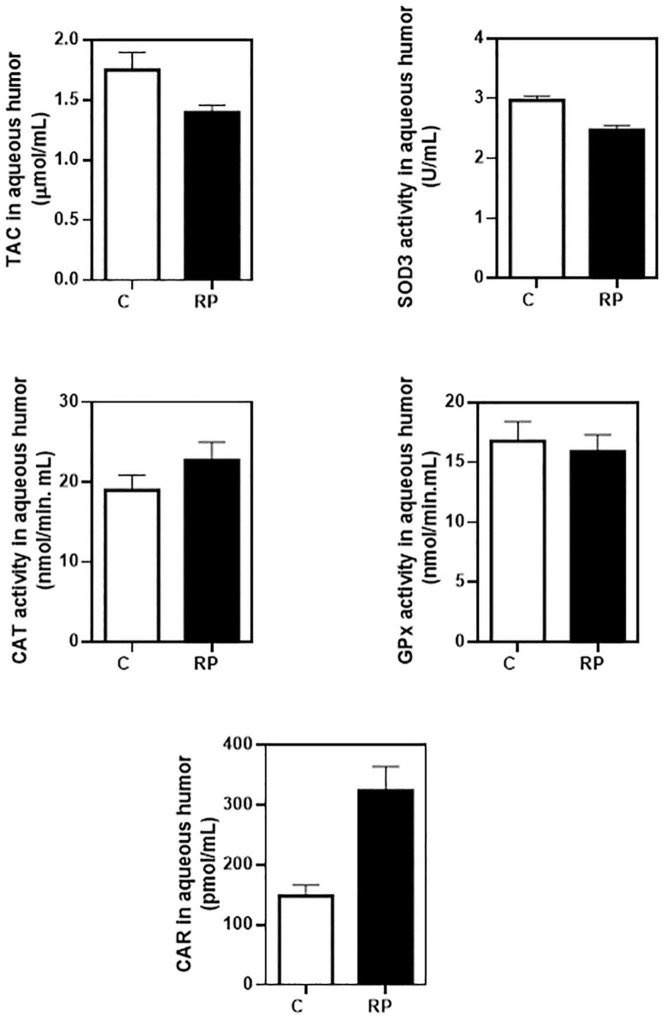
Baseline concentrations of antioxidant and oxidant markers in aqueous humor of controls and RP patients. Ocular redox status was determined by measuring total antioxidant capacity (TAC), extracellular superoxide dismutase (SOD3), catalase (CAT) and glutathione peroxidase (GPx) activities, and protein carbonyl adduct (CAR) content with commercial kits. All data were plotted in bar graph with standard error bars. C, control subjects; RP, patients with retinitis pigmentosa.

The mean baseline level for each parameter is shown in Figure 3 (statistic descriptive). We performed a Bayesian analytical approach to calculate the probability of a relevant difference between RP patients and controls including age and gender as confounding factors. Therefore we indeed controlled the potential imbalances in gender, or age between groups. In aqueous humor the probability that the control group had higher values of TAC and SOD3 activity than the RP group was 99.9 and 99.7%, respectively (Table 3). The probability that the RP group had higher values of than the control group was 97.6%.

**FIGURE 4:**
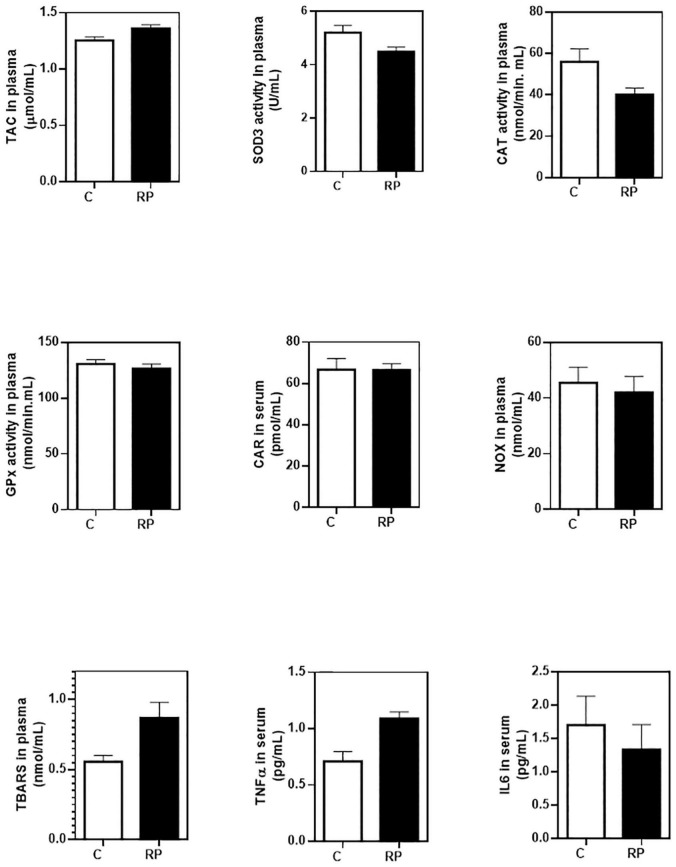
Baseline concentrations of antioxidant, oxidant and inflammatory markers in blood of controls and RP patients. Peripheral redox status was determined by measuring total antioxidant capacity (TAC), extracellular superoxide dismutase (SOD3), catalase (CAT) and glutathione peroxidase (GPx) activities, lipid peroxidation (TBARS formation), nitric oxide metabolites (NOX), and protein carbonyl adduct (CAR) content with commercial kits. Serum cytokines were measured by commercial enzyme immunoassay kits. All data were plotted in bar graph with standard error bars. C, control subjects; RP, patients with retinitis pigmentosa; TBARS, thiobarbituric acid reactive substances; NOX, nitrites and nitrates.

We also measured blood levels of TAC, SOD3, CAT, and GPx activities and CAR, TBARS, and NOX (Figure 4). In plasma samples the probability that the control group had higher values of SOD3 and CAT activities than the RP group was 96.2 and 90.2%, respectively (Table 3). The probability that the RP group had higher values of TBARS, a marker of lipid peroxidation, or TAC was relatively high (85.3 and 88%, respectively).

These data supported the idea of an imbalanced ocular redox status and, to a lesser extent, altered systemic redox status in RP patients that could contribute to visual dysfunction.

As shown in Figure 4 (statistic descriptive) and Table 3, we detected a TNFα upregulation with a very high probability (99.9%) in the group of RP patient. No differences were observed in IL6 concentration.

### Oral NUT Supplementation Delayed Retinal Dysfunction in Patients With Retinitis Pigmentosa

As shown in the flow chart (Figure 1), 31 RP patients were finally enrolled and randomly assigned to NUT (A) or PLC (B) group. Unfortunately, six RP patients were lost to 24 months (19% of the participants) because of pregnancy intention, difficulties to collect the capsules every 2 months or miss taking them.

**FIGURE 5:**
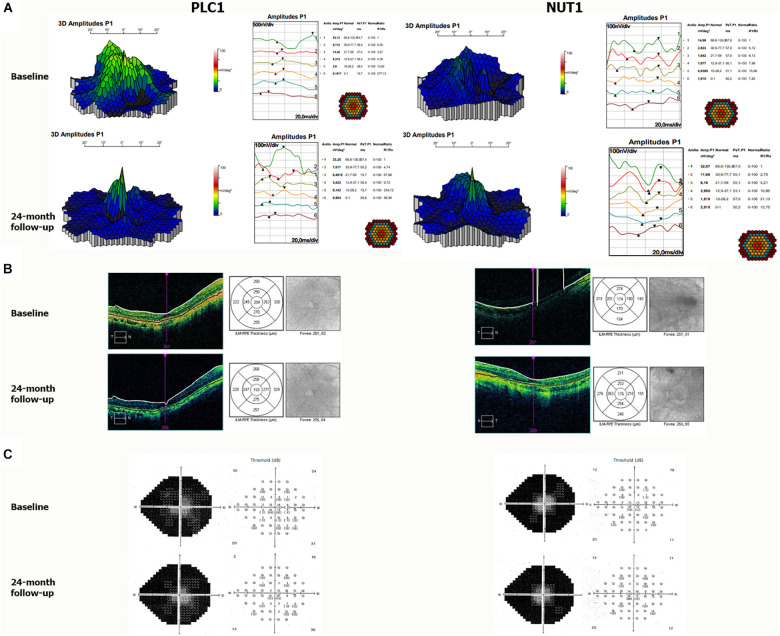
Representative visual outcomes of two patients who received NUT or placebo. **(A)** mfERG tests showing 3D map of P1 amplitudes (right) and ring analysis of P1 amplitudes (left); **(B)** Cirrus SD-OCT analysis showing retinal images and corresponding segmentation map, nine Early Treatment Diabetic Retinopathy Study (ETDRS) regions; and **(C)** automated VF showing grayscale map (right) and threshold (left) of retinal sensitivity in decibel (dB) from the right eye before and after 2 years of supplementation with PLC or NUT. PLC1, patient one with RP who received placebo; NUT1, patient one with RP who received NUT.

The primary outcome was the sum of the P1 amplitudes of the four quadrants change in the central 30° of the retina from baseline to month 24 (mfERG responses). Besides, SD-OCT scans, and automated VFs were evaluated from baseline to month 24. We analyzed the mfERG responses, OCT images and automated VFs on 62 eyes of 31 RP patients at baseline and 50 eyes of 25 RP patients after 2 years of nutritional supplementation with PLC or NUT (Tables 4–7 and Figure 5). We considered the potential influence of age, gender, and ORAC values on the magnitude of change in visual outcomes in the Bayesian analysis. The analysis only considered data from patients who finished the study.

**TABLE 4:** P1 amplitude and implicit time of mfERG recordings at baseline and 24 months after nutritional supplementation.

Multifocal ERG	Q1	Q2	Q3	Q4	Σ Q1–Q4
**P1 amplitude (nV/deg^2^) (*n* = 20 eyes)**					
Baseline NUT Mean (SD)	2.48 (1.76)	2.23 (1.52)	2.59 (1.34)	2.73 (1.73)	10.06 (4.33)
After 24 months NUT Mean (SD)	2.54 (1.43)	3.46 (1.28)	2.80 (1.52)	3.01 (1.53)	11.82 (3.05)
**P1 amplitude (nV/deg^2^) (*n* = 30 eyes)**					
Baseline PLC Mean (SD)	2.66 (2.11)	2.62 (2.32)	2.48 (1.88)	2.61 (1.78)	10.38 (5.91)
After 24 months PLC Mean (SD)	1.86 (1.29)	2.11 (1.44)	2.15 (1.26)	2.83 (1.63)	8.89 (3.80)
**P1 implicit time (ms) (*n* = 20 eyes)**					
Baseline NUT Mean (SD)	38.79 (11.20)	35.18 (10.96)	40.16 (14.30)	39.20 (9.29)	153.3 (30.8)
After 24 months NUT Mean (SD)	36.02 (10.84)	41.15 (7.67)	37.33 (11.67)	33.83 (7.98)	148.3 (26.97)
**P1 implicit time (ms) (*n* = 30 eyes)**					
Baseline PLC Mean (SD)	39.97 (11.28)	36.11 (10.86)	39.10 (11.25)	38.16 (10.29)	153.3 (27.48)
After 24 months PLC Mean (SD)	37.83 (10.22)	34.05 (11.68)	38.69 (9.17)	39.18 (9.88)	149.7 (27.05)

**TABLE 5:** Automated perimetry to assess retinal sensitivity at baseline and 24 months after nutritional supplementation.

Automated perimetry	Σ Q1–Q4
**Threshold of stimulus intensity (dB) (*n* = 20 eyes)**	
Baseline NUT Mean (SD)	267 (46)
After 24 months NUT Mean (SD)	149 (41)
**Threshold of stimulus intensity (dB) (*n* = 30 eyes)**	
Baseline PLC Mean (SD)	273 (57)
After 24 months NUT Mean (SD)	145 (23)

**TABLE 6:** Early Treatment Diabetic Retinopathy Study sector map of macula using CIRRUS software (SD-OCT) at baseline and 24 months after nutritional supplementation.

ETDRS SECTORS	R1	R2	R3	R4	R5	R6	R7	R8	R9	Mean R1–R9
**Thickness from ILM to RPE (μ m) (*n* = 20 eyes)**										
Baseline NUT Mean (SD)	191 (53)	252 (48)	235 (56)	260 (43)	258 (53)	229 (33)	212 (35)	225 (29)	253 (24)	235 (32)
After 24 months NUT Mean (SD)	204 (60)	230 (78)	245 (49)	268 (82)	257 (52)	198 (61)	226 (35)	254 (101)	250 (41)	237 (44)
**Thickness from ILM to RPE (μ m) (*n* = 30 eyes)**										
Baseline PLC Mean (SD)	247 (78)	271 (59)	278 (60)	291 (71)	284 (73)	238 (60)	235 (90)	253 (57)	264 (51)	261 (56)
After 24 months PLC Mean (SD)	234 (57)	262 (48)	252 (60)	271 (61)	279 (47)	235 (56)	230 (90)	243 (77)	260 (39)	251 (41)

**TABLE 7:** Bayesian analysis of visual function in RP patients after 2 years of the nutritional supplementation.

Bayesian analysis at baseline	ES	95% CrI	Pr of PLC < NUT	ROPE	Pr of PLC > NUT	*P*-value
**Visual outcomes (both eyes)**						
P1 amplitude (Σ Q1–Q4)	−0.180	−0.365; −0.004	0.923	0.071	0.006	0.041
Implicit time-mfERG	−0.350	−4.790; 4.140	0.151	0.756	0.093	0.840
VF	−0.139	−0.702; 0.417	0.465	0.396	0.137	0.601
Retinal thickness (SD-OCT) (**Mean R1–R9)**	−1.210	−6.480; 4.110	0.037	0.961	0.003	0.305

*ES, estimate; Crl, credible interval; Pr, probability; RP, patients with retinitis pigmentosa; C, controls; ROPE, region of practical equivalence; mfERG, multifocal electroretinography; VF, visual field; SD-OCT, spectral domain-optical coherence tomography.*

Regarding mfERG responses, the sum of P1 amplitudes for the four quadrant (Q1–Q4) before supplementation was 10.1 ± 4.3 and 10.4 ± 5.9 nV/deg^2^ for the NUT and PLC group, respectively. The sum of the implicit times for P1 amplitudes before supplementation was 153 ± 31 and 153 ± 25 ms for the NUT and PLC group, respectively (Table 4). Two years after the nutritional supplementation the sum of the P1 amplitudes of the four quadrants was also similar to baseline in the NUT group (11.8 ± 3.1 nV/deg^2^) but it got worse in the PLC group (8.9 ± 3.8 nV/deg^2^) (Table 4). The sum of the implicit times for P1 amplitudes after supplementation was similar in both groups (Table 4). As shown in Table 7, the electrical response of the retina measured by mfERG responses evolved worse after 2 years in the PLC group than in the NUT group with a probability of 92.5% (Table 7). Frequentist analysis confirmed this finding (*P* = 0.041).

Patients underwent Humphrey perimetry to obtain automated VF data. At baseline the sum of the four quadrants was 267 ± 46 and 273 ± 57 dB for NUT and PLC group, respectively. Bayesian approach suggested no effect on VFs after nutritional supplementation with NUT or PLC (Table 7).

Macular thickness was measured in nine regions (R1–R9) limited by solid circles of 1, 3, and 6 mm of diameter centered on the fovea by SD-OCT (Table 6). At baseline, the mean macular thickness of all regions was 235 ± 32 and 261 ± 56 μm for NUT and PLC group, respectively. After nutritional supplementation macular thickness was 237 ± 44 and 251 ± 41 μm for NUT and PLC group, respectively. Bayesian approach suggested no effect on the macular thickness (Table 7).

The mfERG findings suggested us a disease stabilization in the NUT group compared to PLC group. However, no effects were observed for SD-OCT nor VF in NUT group.

### Oral NUT Supplementation Ameliorated Redox Status in Patients With Retinitis Pigmentosa

**FIGURE 6:**
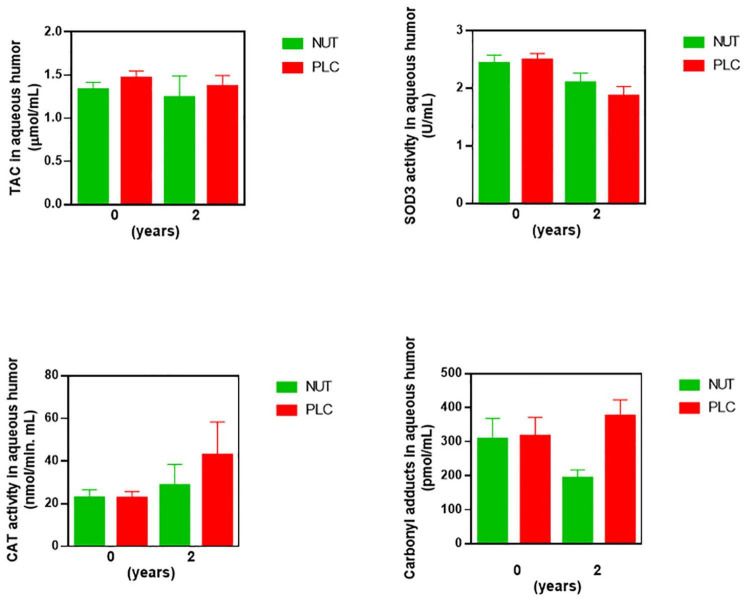
Concentrations of antioxidant, oxidant, and inflammatory markers in aqueous humor of RP patients after 2 years of supplementation. Ocular redox status was determined by measuring total antioxidant capacity (TAC), extracellular superoxide dismutase (SOD3), and catalase (CAT) activities, and protein carbonyl adduct (CAR) content with commercial kits. All data were plotted in bar graph with standard error bars. NUT, patients with RP who daily received a mixture of nutraceuticals; PLC, patients with RP who daily received placebo.

As secondary outcome measures, we assessed redox status and inflammation after 2 years of supplementation. We presented ocular redox status markers at baseline and after 2 years of NUT or PLC supplementation in Figure 6 (statistic descriptive). In aqueous humor GPx activity was not analyzed due to a limited sample availability in some patients. We considered the potential influence of age, gender, and ORAC values on the magnitude of change in biomarker levels in the Bayesian analysis.

The probability that the PLC group presented higher content of CAR and CAT activity than the NUT group in aqueous humor was 97.7 and 90.4%, respectively (Table 8). Besides, the probability that the NUT group showed higher values of SOD3 activity than the PLC group was 95.6%. Therefore, these results suggested that NUT supplementation ameliorated, at least partly, the imbalanced ocular redox status observed in RP patients.

**TABLE 8:** Bayesian analysis of redox status and cytokines in RP patients after 2 years of the nutritional supplementation.

Bayesian analysis at baseline	ES	95% CrI	Pr of PLC < NUT	ROPE	Pr of PLC > NUT
**Ocular redox status**					
TAC	0.163	−0.327;0.637	0.192	0.109	0.669
SOD3	–0.437	−0.862;−0.007	0.956	0.028	0.012
CAT	0.449	−0.172;1.019	0.045	0.050	0.904
CAR	0.591	0.054;1.139	0.009	0.014	0.977
**Blood redox status**					
TAC	0.005	−0.091;0.104	0.371	0.198	0.431
SOD3	0.116	−0.205;0.445	0.193	0.097	0.709
CAT	–0.183	−0.734;0.377	0.687	0.106	0.206
GPX	–0.034	−5.370;5.339	0.167	0.672	0.16
CAR	–0.234	−0.585;0.122	0.875	0.047	0.077
TBARS	0.037	−0.486;0.536	0.345	0.183	0.471
NOX	0.487	−0.524;1.431	0.114	0.084	0.801
**Blood cytokines**					
TNFα	0.229	−0.268;0.733	0.145	0.083	0.711
IL6	0.275	−0.312;0.924	0.102	0.163	0.735

*ES, estimate; Crl, credible interval; Pr, probability; RP, patients with retinitis pigmentosa; C, controls; ROPE, region of practical equivalence; TAC, total antioxidant capacity; SOD3, extracellular superoxide dismutase; CAT, catalase; GPX, glutathione peroxidase; CAR, protein carbonyl adducts; TBARS, thiobarbituric acid reactive substances; NOX, nitrites and nitrate.*

**FIGURE 7:**
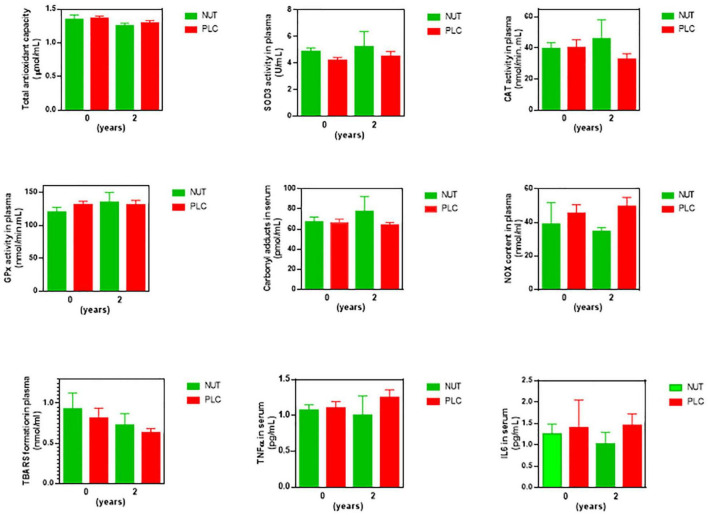
Concentrations of antioxidant, oxidant, and inflammatory markers in blood of RP patients after 2 years of supplementation. Peripheral redox status was determined by measuring total antioxidant capacity (TAC), extracellular superoxide dismutase (SOD3), and catalase (CAT) activities, lipid peroxidation (TBARS formation), nitric oxide metabolites (NOX), and protein carbonyl adduct (CAR) content with commercial kits. Serum cytokines were measured by commercial enzyme immunoassay kits. All data were plotted in bar graph with standard error bars. NUT, patients with RP who daily received a mixture of nutraceuticals; PLC, patients with RP who daily received placebo; TBARS, thiobarbituric acid reactive substances; NOX, nitrites and nitrates.

At systemic level, the supplementation with NUT increased the probability of having higher content of CAR than the supplementation with PLC up to 87.5%. Besides, the probability that the PLC group had higher values of NOX than the NUT group was 80.1% (Table 8). None of the other oxidative stress or antioxidant markers seemed to be affected by the treatments NUT or PLC in blood (Figure 7, statistic descriptive). It seemed that NUT had a slight impact on serum cytokines. The content of TNFα and IL6 were higher in the PLC group than in the NUT group with a probability of 71.1 and 73.5%, respectively (Table 8).

## Discussion

Retinitis pigmentosa encompasses a heterogeneous group of inherited retinal dystrophies characterized by progressive photoreceptor degeneration resulting in visual impairment and even blindness. Several evidence suggested that oxidative stress could negatively influence RP progression.

We previously shown an altered redox status in biological samples of RP patients (11). In this previous study, we evaluated some antioxidant (TAC and SOD3 activities) and oxidant (TBARS and nitrotyrosine) molecules in aqueous humor or blood from RP patients and controls. The findings suggested an unbalanced redox status in RP patients that could contribute to RP progression. Then Campochiaro et al. showed ocular oxidative stress with a reduction of the ratio between reduced and oxidized glutathione (GSH/GSSG), and an increase in protein carbonylation (CAR) in aqueous humor of RP patients. However, they did not observed changes at systemic level (12). More recently, Murakami et al. showed a lower SOD3 activity in RP patients with severe degeneration than in those with mild degeneration. They also suggested a negative relationship between SOD3 activity and VA (46).

In the current study, we confirmed an unbalanced redox status with lower antioxidant response (TAC and SOD3 activity) and higher protein carbonylation (CAR content) in aqueous humor of RP patients. We observed systemic alterations in the antioxidant response including lower SOD3 and CAT activities and higher oxidative damage (TBARS formation) in RP patients than in controls. However, we did not observe lower systemic TAC or higher systemic CAR values in RP patients than in controls. Differences in ocular and systemic TAC values could be due to differences in antioxidant composition in these biological fluids. Aqueous humor is mainly composed of water (around 99.9%) with a small quantity of vitamins, proteins, carbohydrates, growth factors, and cytokines, among others. It contains low molecular weight antioxidants such as ascorbic acid, tyrosine, glutathione, uric acid, or cysteine. Ascorbic acid, a free radical scavenger, is present at high concentrations and contribute up to 73% of TAC in this ocular fluid (47). It seems that ascorbic acid is the main contributor of antioxidant effects in aqueous humor. In blood the most potent free radical scavenger is uric acid which contribute up to 50% of blood TAC (serum) (48). In the current study, we did not evaluate uric acid nor ascorbic acid content in controls and RP patients. It was previously reported that a mixture of antioxidants containing ascorbic acid preserved cone function in *rd10* mice (49). Further studies are needed to better understand the antioxidant compounds which are decreased in RP patients (apart from SOD3 activity).

Protein carbonylation is a harmful irreversible oxidative protein modification which is considered as a major hallmark of oxidative damage. CAR are generated by metal-catalyzed oxidation, lipid peroxidation or glycation (50) and has negative effects on catalytic functions of enzymes (51). For instance, it is suggested that CAR could decrease SOD activity in corticosterone-treated PC12 cells (52).

Currently, there are very few studies evaluating carbonylation in aqueous humor and blood samples. For instance, Hondur et al. showed higher levels of blood and aqueous humor CAR in glaucomatous samples than in controls (53). However, Campochiaro et al. showed high levels of aqueous humor CAR in RP patients and no changes in blood compared to controls, as we observed in the present study (12). RP is an ocular disease thus we expected more prominent oxidative stress-related biomarkers in aqueous humor than in blood. Analysis of aqueous humor is supposed to provide more specific information about RP. The representation of ocular proteins and other markers within the complex and numerous pool of blood proteins is very limited. Therefore, it is not surprising that we did not observe high levels of CAR in serum of RP patients as found in aqueous humor.

We observed elevated serum TNFα levels in RP patients compared to controls suggesting peripheral inflammation. We previously described upregulation of this cytokine in aqueous humor of RP patients (54), corroborating data from other groups. Other authors also detected blood inflammatory markers in RP patients. For instance, serum-high sensitivity C-reactive protein, IL 8 and RANTES were found elevated in blood of RP patients (55, 56). These findings suggest that RP promotes not only ocular but also peripheral inflammatory response. Therefore, it is necessary to study how RP promotes peripheral inflammation.

During recruitment of controls, it was easier to find volunteers for blood collection than for aqueous humor collection. We realize that the recruitment of the same controls would have been a better approach. However, we believe that the observed differences between controls and RP in both biological fluids are consistent with previous works from other research groups (above commented) and our group.

Taking together the above findings in humans and preclinical models, we proposed to perform a nutritional intervention with a mixture of nutraceuticals with antioxidant properties called NUT.

As above commented, nutraceuticals or functional foods are natural substances with health benefits through their antioxidant or anti-inflammatory properties, among others (57). They are very effective antioxidants or/and anti-inflammatory compounds by increasing antioxidant defense, or reducing free radicals or cytokine release (58).

Oxidative damage and inflammation are present not only in RP but also in other retinal diseases such as AMD, or diabetic retinopathy. The use of compounds, e.g., nutraceuticals with antioxidant/anti-inflammatory properties is a very attractive approach for these retinal diseases. Different nutraceutical formulations were assayed in animal models as well as human subjects (59, 60). Omega-3 fatty acids, polyphenols such as resveratrol, carotenoids such as lutein and zeaxanthin, trace elements such as zinc showed neuroprotective effects for the management of diabetic retinopathy, AMD or RP (23, 24, 61, 62).

Goji berry from the plant *Lycium barbarum*, which contains many antioxidant compounds, exhibited neuroprotective effects during AMD, diabetic retinopathy or RP (63, 64). Curcumin, the main component of turmeric, was capable to slow down inflammation in AMD, diabetic retinopathy or RP through the downregulation of IL6, TNFα, etc. (65). Different studies evaluated the effect of the nutritional supplementation with β-carotene, vitamin A, docosahexaenoic acid (DHA), lutein or vitamin E in RP patients. Reviews of these studies revealed that high doses of β-carotene or vitamin A were toxic in some cases (66, 67). Currently, it is unclear whether these compounds could have a relevant beneficial effect for RP patients. More studies were needed to clarify it (66, 67).

In animal models of RP, nutraceuticals such as naringenin, quercetin (21), resveratrol (68), or curcumin (69) also showed a neuroprotective effect on photoreceptor survival and RP progression. Recently, we have demonstrated that oral administration of NUT was capable to ameliorate retinal function, redox status and inflammation in retinas from an autosomal recessive model of RP, *rd10* mouse (26). In particular, NUT ameliorated retinal response to light stimulation (ERG responses), increased TAC, activity of cytosolic SOD (SOD1), and decreased TBARS formation at postnatal day 18, first peak of photoreceptor degeneration (rods) in this model. Besides, NUT reduced retinal inflammation including reactive gliosis, microglia activation and upregulation of the pro-inflammatory cytokines IL6 and IL1β (26).

NUT contains the trace elements copper, zinc, and selenium, vitamins A, B6, and B9 (folic acid) and the carotenoids (xanthophylls) lutein and zeaxanthin. The compounds were selected and combined based on previous studies where they showed protective effects on retinal disorders [reviewed in (26)]. Other formulations including the AREDS and AREDS2 showed protective effects for patients with AMD (70–73). These commercial formulations contain relatively high doses of vitamin C, E, copper, and zinc plus β-carotene (AREDS) or lutein/zeaxanthin (AREDS2) (74, 75).

Many types of nutraceuticals, and studies in animal models and humans for many diseases have been performed. However, the nutraceutical formulations still show a limited efficacy on RP progression. There are several reasons including the lack of well-designed studies based on combined compounds, the scarcity of evidence available on RP compared to other retinopathies (e.g., AMD), the need to characterize the mechanisms responsible for the observed effects, the relatively short duration of the studies, etc.

In the current study, we evaluated the effect of NUT on visual function, redox status and to a lesser extent on inflammation. The primary outcome was the sum of the P1 amplitudes of the four quadrants change in the central 30° of the retina from baseline to month 24 (mfERG responses). mfERG responses reflected that patients who received PLC got worse electrical response than those who received NUT. However, we did not observe differences in VFs nor SD-OCT analysis. RP is a degenerative disease and mfERG responses are decreasing throughout RP progression. Thus we did not expect an improvement but, a delay in the reduction of mfERG responses after NUT supplementation. We observed the natural history of RP progression (related to mfERG responses) in PLC group. However, our results suggested that NUT supplementation reduced the worsening of the electrical response after 2 years.

Multifocal electroretinogram is a technique that provides a topographical map reflecting retinal function. It can detect focal changes in the retinal function as RP progresses (42, 76). The mfERG technique is an objective outcome measure in treatment studies involving patients with retinopathies such as RP. To measure VFs we performed a computerized VF perimetry. During this procedure the eye was fixed on a reference point and patient had to respond to different light stimuli. It was a subjective test because it required compliance of and concentration of the patient (77). The subjective nature of this test and the follow-up of the study limited to find differences in this ophthalmologic parameter.

Traditionally tests for following the progression of visual function during RP included VA, Ganzfeld ERG or mfERG and VFs (78). While the concentric VF loss is more progressive, the loss of VA gradually decline with age in RP patients. However, the measurements of both tests (VF and VA) are variable. As above described, these tests present a high subjectivity, low test-retest reliability and increased variability as severity of disease increases (79).

We did not observe a beneficial effect on macular thickness measured by SD-OCT. SD-OCT is a non-invasive technique to obtain cross-sectional retinal images by differential near-infrared light reflection at optical interfaces. It is a well-established method that helps to evaluate the morphological changes related to retinal function in RP patients (80). We were not surprised with the results of SD-OCT analysis. We could expect a functional impairment but, not significant morphological changes after 2 years of NUT supplementation. We believe that a longer follow-up would be needed to detect differences in VF or structural changes by SD-OCT.

Other nutraceuticals ameliorated retinal function in RP patients or animal models. For instance, chlorogenic acid, a polyphenol, improved mfERG responses after 3 months of supplementation in RP patients (81). Chan et al. showed that *L. barbarum* supplement delayed cone degeneration by ameliorating the loss of VA (82). Lutein induced a short-term vision improvement in RP patients (83, 84). In *rd10* mice, supplementation with naringenin plus quercetin or with lutein plus zeaxanthin slowed down retinal degeneration and oxidative stress (21, 85).

In the current study, key secondary outcomes included mean change in redox and inflammatory markers from baseline to month 24. After 2 years of supplementation, the decrease of ocular SOD3 activity was lower in RP patients who received NUT than those who received PLC. Besides, the nutritional supplementation with NUT ameliorated CAR in aqueous humor and tended to reduce serum cytokines IL6 and TNFα. We do not know how the nutraceutical mixture reduced CAR in aqueous humor. We speculate that NUT could act as free radical scavenger or as modulator of other cellular antioxidant systems. For instance, it is suggested that lutein and zeaxanthin prevented protein carbonylation by increasing GSH levels in human lens epithelial cells exposed to H_2_O_2_ (86). More recently, vitamin B6 supplementation reduced protein carbonylation in kidney, pancreas and liver in alloxan induce diabetic rats (87). Maybe reduced protein carbonylation was responsible for a better SOD3 activity in aqueous humor of patients treated with NUT.

As previously commented, we were not able to analyze GPx activity in aqueous humor after supplementation. We collected between 100 and 125 μL of aqueous humor but, we obtained less amount for some patients. Therefore, we did not measure GPx activity (15 μL/samples), and assess the effect of supplementation on this antioxidant enzyme.

Improvement on redox status and/or inflammation after nutraceutical supplementation was previously described. For instance, supplementation with saffron or ellagic acid, a polyphenol, improved systemic antioxidant response (TAC, SOD, and GPx activities) in patients with ulcerative colitis or with type 2 diabetes, respectively (88, 89). Vitamin D or alpha lipoic acid supplementation increased antioxidant response (SOD and/or GPx, CAT activities) and reduced oxidative damage (MDA) in hemodialysis patients (90, 91).

In Ophthalmology, alpha lipoic acid supplementation improved serum SOD activity in AMD patients (92). Supplementation with high dose DHA showed beneficial effects for patients with diabetic retinopathy, glaucoma and macular edema (93). Specifically in glaucoma, DHA supplementation increased TAC and decreased MDA and IL6 (94). In diabetic retinopathy combination of DHA with xanthophylls increased TAC, macular function and reduced IL6 (95). AREDs formulation also reduced some inflammatory cytokines including IL6 and TNFα in AMD patients (96).

In our study, NUT supplementation had a lower impact on blood redox status. It seemed that NUT reduced NO metabolites (NOX) but, increased CAR content in blood. Protein carbonylation is an indicator of the accumulation of oxidative damage in protein for a long period. We were not able to explain this increase after NUT supplementation.

Apart from evaluating redox status, inflammation and visual function, we estimated dietary intake of several nutrients with antioxidant properties. We observed similar estimated dietary intake for the majority of nutrients except for selenium in RP patients. Selenium is a trace element essential for selenoproteins such as GPx. Selenoproteins have antioxidant properties preventing from free radical formation (97, 98). Selenium deficiency could have an adverse effect on health. Dietary Se deficiency affects 0.5–1 billion people in the world (99).

However, serum selenium concentration did not reflect this difference in estimated dietary intake. Both groups presented similar blood values. This effect was, also, observed by Dias et al. (100) indicating that body selenium did not reflect a relationship with Se intake. In fact, Thomson (101) showed that blood Se concentrations varied with different forms of dietary selenium and, perhaps, among different population groups. In any case, estimated Se intake in RP patients was within the dietary intake recommendations (average 60 μg per day for men and 53 μg per day for women) (102).

We also detected higher estimated ORAC score and blood lutein and zeaxanthin concentrations in RP patients than in controls. The fat-soluble xanthophylls are essential macular pigments. They protect retina from oxidative damage by quenching free radicals (103). These findings could suggest that RP patients had a more antioxidant diet. We were not able to detect the water-soluble vitamins B6, and folic acid (vitamin B9) in blood by UHPLC-MS/MS analyses. B6 and B9 vitamins are dissolved in water and rapidly absorbed into tissues for use. They are not stored in the body, and in excess they are quickly excreted in urine. Maybe our samples did not contained enough amount of these vitamins to detect by UHPLC-MS/MS.

Currently, RP has no treatment except for RPE65 gene. Maybe patients were more prone to have healthy habits with a more balanced diet than controls. However, in 2016 Sofi et al. analyzed the dietary profile of patients with RP and Stargardt’s disease and compared it with the DRI from the Italian Society of Human Nutrition. The authors concluded that a high proportion of patients had a hyper-lipid diet with a low intake of fiber. Besides, vitamin A intake was low in RP patients (104). More studies are needed to clarify the dietary profile of RP patients in Spain.

Our study present some limitations. For instance, we observed a relatively high rate of attrition, thus increasing the potential for bias in our analyses. However, almost all of the dropouts can be considered as MCAR. Considering this, and that our modeling strategy was based on linear mixed models, we did not expect a significant amount of bias in our estimates according to the results presented by Twisk et al. (105) and also by Bell and Fairclough (106). We are aware of the small sample size of this study and that this limitation could affect the precision of the estimates from our models (but not the validity of our results). For that reason, we propose additional studies enrolling more participants to support our current findings. We were not able to measure (sample availability) the same markers in ocular and systemic samples (e.g., TBARS or TNFα) or others such as GSH. Finally, we understand that monitoring compliance by indirect methods could be a limitation because they could overestimate adherence.

## Conclusion

We are aware of the limitations of this study (e.g., number of participants, amount of ocular samples, withdrawals, indirect methods of monitoring compliance, different controls, or TBARS determination instead of MDA determination). However, we highlighted that NUT supplementation achieved better electrical responses to light stimuli (mfERG responses) in the retina than PLC supplementation. NUT supplementation could be useful, at least partly, for delaying retinal degeneration in patients. Besides, NUT supplementation ameliorated ocular redox status by decreasing CAR and improving SOD3 activity (compared to PLC group). Collectively, evidence of this study suggests that NUT supplementation could be useful for protecting retina from retinal degeneration by slowing down visual dysfunction and ameliorating unbalanced redox status in RP patients. However, additional studies with more participants, other redox markers and a longer follow-up period would help to confirm these findings.

## Data Availability Statement

The original contributions presented in the study are included in the article/supplementary material, further inquiries can be directed to the corresponding author.

## Ethics Statement

The studies involving human participants were reviewed and approved by the Medicaments Research Ethics Committee (CEIm) of La Fe University Hospital (Valencia, Spain). The patients/participants provided their written informed consent to participate in this study.

## Author Contributions

LO-G, SV, and IC performed the biochemical determinations. LO-G, DS, JSC, JM, and RR contributed to conception and design of the study. JSC designed the formula. LO-G and RR organized the database. DH and RR performed the statistical analysis. DS and EG-G performed the ophtalmic examinations. DS carried out sample collection and follow-up of the patients. NM-C and BM performed the dietary intake analysis. JM-T supervised dietary intake analyses. MM prepared the nutraceuticals. NV-C and EL-B supervised the preparation of the nutraceuticals and randomization. RR wrote the first draft of the manuscript. DS, LO-G, EG-G, DH, JSC, and RR wrote sections of the manuscript. All authors contributed to manuscript revision, read, and approved the submitted version.

## Conflict of Interest

The authors declare that the research was conducted in the absence of any commercial or financial relationships that could be construed as a potential conflict of interest.

## Publisher’s Note

All claims expressed in this article are solely those of the authors and do not necessarily represent those of their affiliated organizations, or those of the publisher, the editors and the reviewers. Any product that may be evaluated in this article, or claim that may be made by its manufacturer, is not guaranteed or endorsed by the publisher.

## Footnotes

1
www.randomization.com
